# Discrepancy between subjective perception and objective cognitive performance in attention assessment within the winners project for cancer survivors. a case report

**DOI:** 10.1192/j.eurpsy.2024.1346

**Published:** 2024-08-27

**Authors:** C. Gonzalez-Perez, E. Moran, N. Malpica, J. Alvarez-Linera, H. Melero, M. Alonso, M. Esteban, A. Perez-Martinez, E. Fernández-Jiménez

**Affiliations:** ^1^Department of Pediatric Hemato-Oncology, La Paz University Hospital; ^2^Children Psychology, Juegaterapia; ^3^ Medical Image Analysis and Biometrics Laboratory (LAIMBIO), Rey Juan Carlos University; ^4^Department of Neuroradiology, Hospital Ruber Internacional; ^5^Psychobiology and Methodology in Behavioral Sciences, Complutense University; ^6^Juegaterapia; ^7^Department of Psychiatry, Clinical Psychology and Mental Health, La Paz University Hospital, Madrid, Spain

## Abstract

**Introduction:**

Paediatric cancer survivors have a risk for neuropsychological impairment due to the disease and the treatment received. These affections have been neglected in the follow-up of these patients. It is important to identify the most valid outcomes in the evaluation of neurocognitive sequelae in childhood cancer survivors.

**Objectives:**

This work aims to compare the results obtained between subjective perception of caregivers and objective cognitive performance based on validated attention tests.

**Methods:**

In a randomized controlled and unblinded trial to demonstrate the benefit of video games on different neurocognitive areas in cancer survivors, we studied attention functioning before and after the intervention program. The attention deficit subscale from the Behavior Assessment System for Children 3rd edition (BASC-3), self- and parent-reported versions, and the Continuous Performance Test, 3rd edition (CPT 3) will be used as outcomes (z scores: mean = 0, S.D. = 1).

**Results:**

We observed an improvement in attention after intervention using the CPT-3 (omissions z = 1.2; hit reaction time z = 3.4; hit reaction time block change z = 1.2 versus hit reaction time z = 3.6 without other atipycal z scores after intervention), changing the attentional pattern from “ADHD” to “slowed”. However, in the parent-reported version of the BASC-3, a worsening in the attention subscale is observed (z = 0.3 pre-intervention vs z = 1.0 post-intervention) while the self-reported version of the patient didn’t show any significant changes (z = 1.4 pre-intervention vs z = 1.1 post-intervention).

**Conclusions:**

It is essential to use objective tests to measure neurocognitive sequelae in these patients. Subjective surveys can provide additional information, but not substitute the above.

**Disclosure of Interest:**

None Declared

